# Geographical equations of *Swertia mussotii* bioactivities: evidence from the western Sichuan region of China

**DOI:** 10.3389/fpls.2023.1112164

**Published:** 2023-06-28

**Authors:** Xiaobo Wang, Cheng Shen, Tao Chen, Xiaodan Zhou, Yulin Li

**Affiliations:** ^1^ Northwest Institute of Eco-Environment and Resources, Chinese Academy of Sciences (CAS), Lanzhou, China; ^2^ Northwest Institute of Plateau Biology, Chinese Academy of Sciences (CAS), Xining, China; ^3^ Department of Pharmacy, Gansu Provincial Hospital, Lanzhou, China

**Keywords:** *Swertia mussotii* Franch, environmental factor, bioactive compounds, correspondences analysis, Western China

## Abstract

*Swertia mussotii* is the most authentic raw material used in Tibetan medicine in China for its various bioactivities. This natural medicine resource is at risk of being exhausted due to the double interference of climate change and anthropogenic over-collection. Little is known about habitat characteristics and the crucial environmental factors that influence the levels of active ingredients. The goal of this study is to understand the variability in the bioactive compound content of a wide range of wild *S. mussotii* as it adapts to changing environmental conditions. The target compound content of the whole plant material was analyzed with the environmental explanatory variables of the field sample sites using a constrained ordination method for their correlation analysis. The results show that 16.3 percent of the sampled wild *S. mussotii* populations with the highest bioactive content can be grouped into the elite type. The most prominent environmental variables affecting the content of major bioactive products include altitude, aspect, soil TK content, Fe content, and C/N and N/P ratios. Altitude and aspect put indirect effects that are mediated by plant height and density, N/P ratio puts a direct effect, while soil TK content, Fe content and C/N ratio have both direct and indirect effects on the bioactivity of *S. mussotii*. In addition to the total negative effects of altitude and C/N ratio, the remaining factors play a driving role. These findings demonstrate variation by geographical conditions across S. mussotii accessions for physiologic responses and secondary compounds in wild populations. The knowledge gained from this study can be used for environmental and plant physiology research, efficient collection of naturally active compounds, and conservation strategies for rare natural plant resources.

## 1 Introduction

In nature, various plant secondary metabolites (SMs) mean that many plants containing large amounts of these bioactive compounds have long been used as important sources of traditional medicines, industrial raw materials, and spices (Li and Vederas, 2009; Piccolella et al., 2018). Offering great potential for their nutraceutical and medicinal exploitation, the extraction and purification of SMs from wild natural plants usually not only requires a complex and time-consuming process, but also obtains quite a low yield of the target product (Christensen, 2020; Tava et al., 2022). Geographical locations have an important effect on the quality of many medicinal plants because environmental abiotic factors are always regulating the formation and storage of SMs in plants (Szakiel et al., 2011). Therefore, finding reliable ways to enhance this systematic process to increase the yield of natural compounds has been a serious long-term challenge for researchers (Ashraf et al., 2018). This strategy also holds extremely important prospects for the development of improved quality and conservation of traditional Chinese medicinal plants.

To date, much research has focused on finding the most productive plant species and optimizing breeding conditions to improve the output of plant SMs (Motta et al., 2019; Huang et al., 2022). While the number of studies directly highlighting the significant correspondence between abiotic factors and wild-type plant SMs is growing, the current understanding of the different geographical factors that regulate the production of active metabolites in diverse plants is still limited. In literature, materials sampled from the South-Central U.S. has 7-fold lower mitragynine content than the Southeast Asian samples (León et al., 2009), and there is also a significant content difference between Thai origin trees and Malaysian ones (Leksungnoen et al., 2022). Even on a regional scale, the active product contents of medical materials vary enormously depending on where they come from (Lu et al., 2009; Yang et al., 2010). That means not only that abiotic stresses are interrelated, but changes at the physiological, biochemical, and molecular levels are linked, both individually and in combination (Bita and Gerats, 2013; Misra et al., 2021). Therefore, the synthesis and accumulation of phytochemical components are heavily dependent on various environmental conditions of the habitat (Hinojosa et al., 2018; Tabatabaei et al., 2022).

Different combinations of abiotic external conditions regulate the level of variation in the chemical properties of the bioactive compounds that play a decisive role in plant growth. This internal variation is also reflected to some extent in the plant heights and population densities that are directly visible from the outside, indicating a high degree of ecological plasticity. Many climate trends are commonly associated with altitude (Guevara-Terán et al., 2022), which is also an easily identified geographical factor (Sun et al., 2021). A review on the adaptation of medicinal plants to various environmental stresses in the Himalayas provides relatively comprehensive information, in which only one reference is made to the physiological differentiation caused by adaptation to solely different altitudes (Pandey et al., 2019). This means that altitude usually coordinates the influences of abiotic factors, for instance, different altitudes are associated with various annual temperatures (Ahmed et al., 2019). At different altitudes, plants accumulated different SMs levels mainly regulated by changes in temperature and solar radiation (Senica et al., 2016). The way altitude affects plants can also be directly or indirectly through reduced atmospheric pressure (Arce et al., 2021), significantly related to site characteristics such as slope (Mullin et al., 2021). Additionally, climate change that manifests as warming takes contradictory effects on the SMs production of medicinal plants (Holopainen et al., 2018).

*Swertia mussotii* Franch (*S. mussotii*) is a star species of the Swertia genus in the Gentianaceae family growing in the West China mountains. For thousands of years, it has been used in the treatment of liver disorders and jaundice, and has anti-inflammatory, antipyretic, and gallbladder- and diuretic-promoting functions, making it a raw material for a variety of liver and gallbladder diseases (Chen et al., 2015). The bioactive SMs of *S. mussotii* include swertiamarin, gentiopicroside, sweroside, mangiferin, and isoorientin (Tan et al., 2017), applied for dispelling heat and improving gallbladder function, dehumidifying and detoxifying, soothing the liver and strengthening the stomach, helping heart function and nourishing the blood, and are considered as important indexes to measure the material quality (Shen et al., 2021). However, in previous studies on the difference in compound content, scholars tended to make cross-regional and large-scale comparisons in order to emphasize the heterogeneity of site environmental conditions (Bazargani et al., 2021). They did not systematically design environmental variables, so only a qualitative understanding of the quality differences between different places of medicinal materials could be proposed (Lu et al., 2009; Yang et al., 2010). As a result, current knowledge of the interplay between *S. mussotii* quality and environmental variability is not sufficient to aid conservation and rational development of this valuable natural resource.

Taking the homogeneity of geographical units on the macro level into consideration, the objectives of this study include: 1) To examine the correlation between the chemical types of wild *S. mussotii* population and its growing environmental conditions; 2) To identify the classification of active SMs and the sorting of main environmental factors of *S. mussotii* under current climatic conditions; and 3) To clarify the influence mechanism of geographical factors on natural *S. mussotii* growth and its medicinal quality. In this study, we established an HPLC chromatogram of *S. mussotii* extraction to assess the content of plant active ingredients, used unconstrained ordination analysis to assess the overall differences in phytochemical types, screened environmental predictors with constrained ordination for explanation and contribution, and explored the effects and pathways of important influencing factors by regression analysis and structural equation model. This study thus endeavors to use the newly-acquired knowledge to provide a scientific reference for the conservation introduction and exploitation of *S. mussotii* to facilitate the development of appropriate sustainable management measures in future warm and humid climate scenarios.

## 2 Materials and methods

### 2.1 Plant sampling design and field collections of sample material

**Figure 1:**
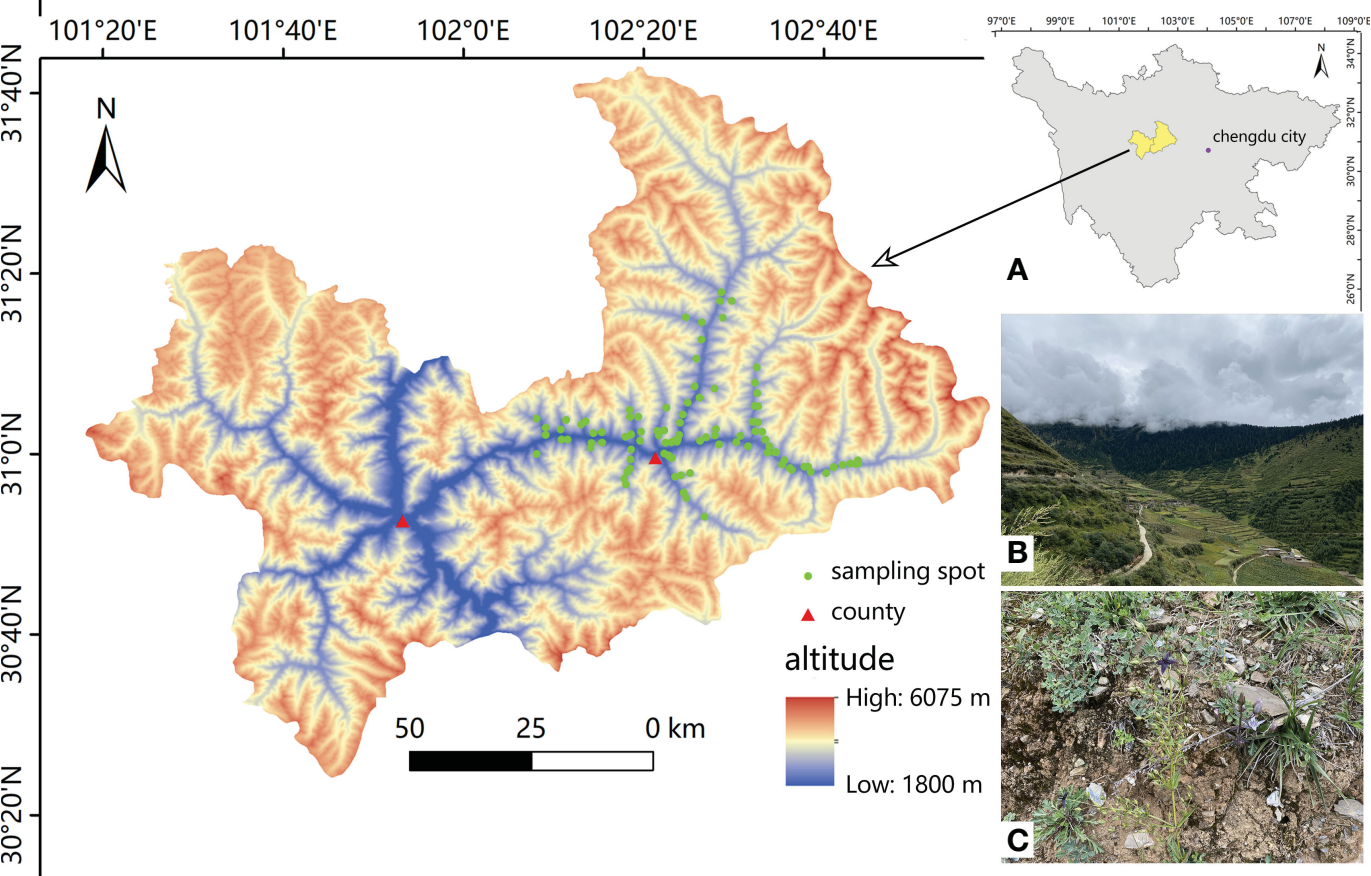
The geographic distribution of the natural population of *S. mussotii* in this study (*n*=98). **(A)** The study area in its administrative geography of western Sichuan province; **(B)** environmental conditions of *S. mussotii via* landscape; **(C)** a typical site habitat of wild *S. mussotii*. Both photos were taken on 31 August, 2021.

A total of 107 potential sites were visited with a local guide, and 98 of these sites with *S. mussotii* growth were recorded for sampling in Xiaojin County, West Sichuan, China in late August 2021. The whole sampling period lasted for 10 days and collections from each sampling site were taken at least 1.5 km apart. According to the intuitive perception on the growth density of S. mussotii found, the dense and sparse groups of study areas accounted for 39.79% and 26.53%, respectively. ArcGIS 10.2 (Esri, Redlands, California USA) was used to construct the location occurrence point map, as shown in **Figure 1**. Wild *S. mussotii* at each sampling site were mostly in full bloom. Flowering adults were thus selected for collection whole, until approximately 20 sample plants were collected within an area of approximately 20 m by 20 m at each sampling point. The average plant height of each collected sample was then recorded. At the same time, the topsoil was removed from the collection area according to the five-point sampling method, and 5-20cm of soil depth was excavated with a sampling shovel, mixed, and marked for preservation.

The stoichiometric analysis was carried out at the institutional center of core facilities and technologies in Northwest Institute of Plateau Biology, CAS following established methods to detect 14 soil characteristics. Among them, Ca, Fe, Mn, Zn, Na, total phosphorus (TP), and total potassium (TK) were analyzed by ICP-MS (Thermo fisher, USA); total salt content (water-soluble cation and anion), available nitrogen (AN), available phosphorus (AP), available potassium (AK), and pH by water solution method with corresponding detectors; and total nitrogen (TN) and organic carbon (C) by elemental analyzer (Elementar, Germany). Then the ratio of carbon to nitrogen (C/N) and nitrogen to phosphorus (N/P) indexes of soil samples could be calculated. The GPS and altitude information was recorded at the center of the sampling site, and the major aspects were recorded under the classification of the eight directions. These obtained data were stored in IBM SPSS 23 software for processing and analysis.

### 2.2 Separation and collection of bioactive products

**Figure 2:**
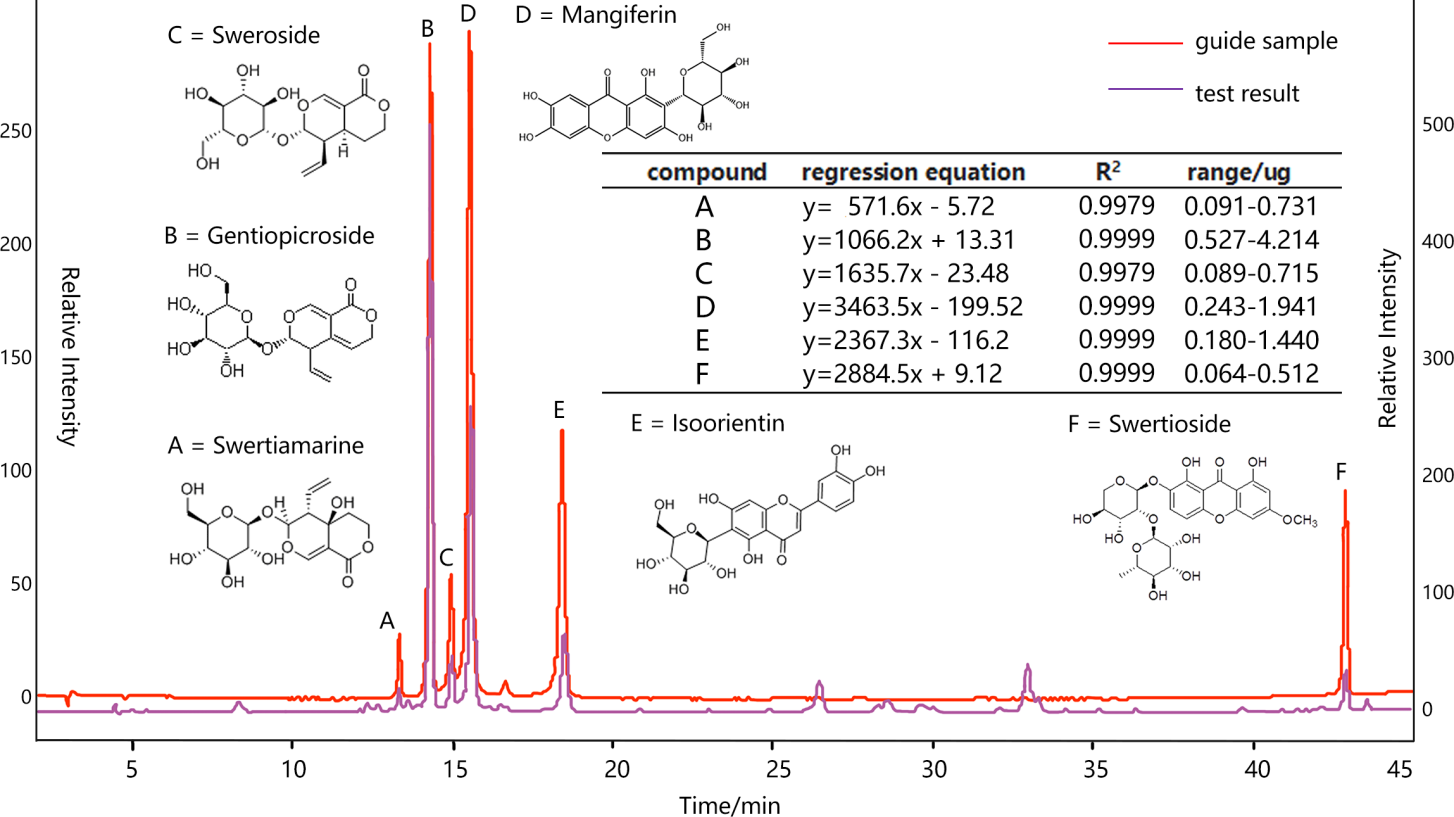
HPLC analysis of ethanol extract of *S. mussotii* conditions: column, DIKMA platisit (250×4.6mm, i.d. 5μm); column temperature, 30 °C; mobile phase, 0.1% glacial acetic acid (solvent α) and CH_3_OH (solvent β); HPLC analysis, 0-45 min, linear gradient from 10% to 40% β during 0-10 min, 10-15 min 40%-42.5% β, 15-25 min 50% β, 25-35 min 60% β, 35-45 min 95% β; flow rate, 1.0 mL/min; detection wavelength, 254 nm.

In view of the processing and application principle of *S. mussotii*, this study focused on swertiamarine (A), gentiopicroside (B), sweroside (C), mangiferin (D), isoorientin (E), and swertioside (F). The content of these compound groups was also the main standard to measure the quality of this herbal medicine in the Chinese medicinal industry (Shen et al., 2021). All samples were naturally shade-dried and crushed, then screened by 60 mesh. Reflux extraction was used and the extraction conditions were as follows: extraction solvent, 30% ethanol; liquid-material ratio; extraction temperature, 80°C; extraction, three times; each extraction time, 90 min. The extraction was titrated to 100 mL for sample injection after filtration. On the other hand, the compounds A~F with different weights of the standard product were fully dissolved in the methanol solution and well shaken at a constant volume, so that the standard solution was configured in a ratio of 1:5:2:2:1:1. Then, 2, 4, 8, 12, and 16 μL of standard solution were injected into HPLC for testing, respectively, to draw a standard curve. The fitted peak area (y) and injection volume (x) were used as regression curves, as shown in **Figure 2**.

The stability, accuracy, repeatability, and addition of standard recovery of the bioactive compound separation and collection processes have been adopted to verify the compound determination. Specifically, measuring the same sample solution at 0,2,4,8,12 and 24 h, respectively, the results showed that the HPLC peak areas RSD of A-F are all less than 3.0%, indicating that the samples are stable at 24 h. Taking 10 μL of the mixed reference solution and sample solution to measure each peak area six times, the former RSD is less than 2.0% and the latter is less than 2.5%, meaning that the instrument and injection process is very precise. Six raw material samples are randomly taken and filled into the same solution volume, and their determinations proved to be similar, with the peak area RSD of all six compounds being less than 3%, suggesting that the method is reproducible. Finally, adding reference solutions with 80%, 100%, and 120%, respectively, corresponding compound contents into a tested sample solution, the new mixed solutions are injected into HPLC to measure the six objective recoveries (n=3) ranging from 98%-103%, with all RSD less than 3%.

### 2.3 Bioclimatic data and data processing

Bioclimatic variables (BIO1-BIO19) were extracted from monthly data from the World Climate Database covering 1950-2000 (www.worldclim.org) (Hijmans et al., 2005) according to the longitude and latitude coordinates of each collection locality; the spatial resolution was 30-arc second, which was robust multivariate bias corrected by meteorological data from 2001-2020 from national weather stations (Chen et al., 2022). Although the sampling sites are not less than 1.5 km from each other, some spots still fall within the same 1 km by 1 km grid as reflected in the GIS. In this case, there are 7 collection points in the same grid that are processed by averaging the data obtained at the two nearest sites.

To study the effect of other climatic conditions, altitude, slope, slope aspect, mean solar radiation intensity, water vapor pressure, and mean wind speed variables were also extracted from the climate dataset. In the assignment of aspect, 1~5 represents north, northwest or northeast, east-west, southwest or southeast, and south respectively, replacing the original 0~360° azimuth values. The altitude result showed a significant correlation with the field GPS records of the sampling points, the correlation coefficient was 0.770 (*p* < 0.001), and the correlation coefficient between the extracted aspect information and the manual records was 0.530 ((*p* < 0.001). This indicats a good reliability of the extracted data.

### 2.4 Statistical analysis

Following the principle of parsimony, the above 41 environmental factors were input into Canoco5 software to test the arch effect of environmental predictors, and the analysis model was simplified by excluding multicollinearity and environmental variables with low contribution (ter Braak and Smilauer, 2012). A factor analysis of 41 environmental variables reveals the influence of more than 4 components, so directly casting constraint ordination method inevitably decreases the level of interpretability. Thus, 18 collinear variables have been removed in data preprocessing based on the correlation of the variables and the contribution of the control variables. The variables with less indicative values than the overall mean value were then eliminated by unconstrained ordination analysis, and the final 13 most informative variables were selected, as shown in **Table 1**. This stepwise analysis also assessed the differences in eigenvalues between environmental variables and bioactive compounds to determine whether erroneous variables or too many variables had been deleted from the environmental dataset.

**Table 1:** Summary of the final environmental variable set of *S. mussotii*. population.

	max	min	mean	stand. dev.	inflation factor
Altitude(m)	3323	2434	2855	182.7	2.56
Aspect	5	1	3.48	1.198	1.86
C/N ratio	10.77	1.38	6.06	1.945	2.51
TP(g/kg)	0.97	0.27	0.57	0.129	1.84
N/P ratio	13.92	2.91	7.67	2.372	1.95
TK(g/kg)	25.7	13.3	18.35	2.371	2.48
Fe(g/kg)	34.4	23.8	29.17	2.317	2.82
Mn(g/kg)	0.91	0.29	0.54	0.084	2.16
Na(g/kg)	12.9	5.36	8.79	1.4	1.59
Zn(mg/kg)	110.3	58.3	80.3	8.371	1.85
TS(g/kg)	2.3	0.6	1.2	0.335	1.59
AK(mg/kg)	283	32.6	111.04	53.019	1.85
MTCM(°C)	-4.6	12.8	-8.26	1.604	3.28

aspect was assigned in 1 to 5 for shady to sunny orientation, respectively; all soil element contents were tested by dry weight; TK and TP refer to total K and P content in soil samples, TS means total soluble salt content, AK refers to available K; MTCM means minimum temperature of coldest month.

The levels of compound content obtained from *S. mussotii* can be distinguished using the clustering method. For example, the significant classification results of K-means clustering indicate that there is a significant difference between elite and common groups in wild populations. In addition, the environmental factors affecting the active compound content can be fitted by regression models. But it is necessary to compare and check the standardized effects of different environmental factors in the structural equation model. The structural equation model in this study was implemented by MPLUS 8 software (Muthén and Muthén, 1998–2017).

## 3 Result

### 3.1 Descriptive statistics of six bioactive compounds

**Figure 3:**
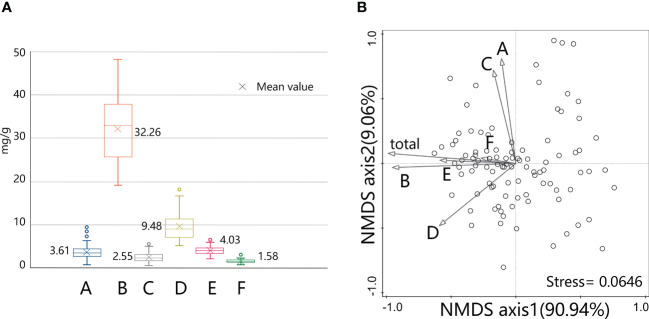
Content levels of active compounds extracted from *S. mussotii* in this study **(A)** and their clustering analysis by NMDS method **(B)**.

According to the extraction scheme in this study, the sum of 6 active compounds in *S. mussotii* from 98 collection sites is 76.75 ± 10.14 mg/g. Compound B as the second peak in the HPLC chromatogram reached the highest content of them all, with an average ratio of over 60%. This is because the separation and collection method employed in this study have been developed to determine the amount of compound B in wild *S. mussotii* material to the greatest extent for the approval of a national standard. Compound D, which averages 17.73% in this work, is a common flavonoid with a wide range of plant sources. The other 4 active constituents each make up less than 10% of the total extracted content, as shown in **Figure 3A**. We put the content data into a non-metric multidimensional scaling (NMDS) analysis, with the ordination model set as 2 dimensions for fitting; the result showed that these two axes have reached 100% of explanatory power on the correlation among active constituents with its stress value returns 0.0646, showing that the model is significant. The arrow directions of different compounds in **Figure 3B** indicate that there is a certain correlation between several components, such as compounds A and C, and B and E. Compound B has contributed the most to the total content of all six for these two vectors in **Figure 3B** are similar, with a *Pearson* coefficient reaches 0.936 (*p*<0.001). At the same time, the compounds D and E also exceed the total content by a coefficient of 0.5, respectively.

Using the contents of the compounds A-F as variables, the 98 existing sets of samples can be grouped by K-means clustering under the iterative classification algorithm. Due to the different content of bioactive compounds, the study sample can be divided into low, middle, high, and elite groups, which contain 26, 27, 29, and 16 populations, respectively. The variance test of the cluster analysis result shows that compounds B, D, and E significant differences in high and low content among the 4 groups, revealing that these 3 compounds are the main indicators in the classification. This can also be understood from the principle of the minority subordination to majority, since the absolute concentration of the three is generally higher. Taking compound B into account, the cluster centers were 43.79mg/g for the elite group and 36.54mg/g for the high group, both greater than the sample mean of 32.26mg/g. Besides, the sequence of cluster centers of compound A and C is inconsistent, which verified the difference from these two compounds to the other in **Figure 3B**. Cluster analysis also suggests that there is an elite type in the wild *S. mussotii* population, which may accumulate higher SMs than other plants.

In terms of plant population and height of growth, the average plant height of *S. mussotii* is 65.7 ± 11.6 cm. The plant height is significantly affected by altitude and slope aspect, with correlation coefficients of -0.509 (*p*<0.001) and -0.666 (*p*<0.001), respectively. That is, *S. mussotii* plants grow shorter overall with increasing altitude, and shorter in the direction of the sun. At the same time, plant height is positively affected by temperature factors, but negatively by precipitation. It is also negatively correlated with the total content of extracted active ingredients with a coefficient of -0.674 (*p*<0.001). On the other hand, the degree of population density recorded by sampling shows a negative significant correlation with altitude to the coefficient of -0.511 (*p*<0.001), while a positive correlation with aspect to the coefficient of 0.348 (*p*<0.001). At present, the population density also reflects the difference in bioactive compound content to some extent, and the correlation coefficient between them is 0.518 (*p*<0.001), showing a significant negative correlation with plant height with a coefficient of -0.208 (*p*=0.040).

Based on the classification results of different groups with high to low bioactive compound content, the independent sample Kruskal-Wallis test was further conducted for 13 key environmental variables extracted from the post-redundancy analysis. The result shows that significant differences occur in aspect (*p*<0.001), soil TK content (*p*<0.001), Fe (*p*<0.001), C/N ratio (*p*=0.007), minimum temperature of coldest month (*p*=0.007), Na (*p*=0.012), and N/P ratio (*p*=0.020), while there is no highlighted difference in soil total soluble salt content (*p*=0.097), AK (*p*=0.111), altitude (*p*=0.118), TP (*p*=0.165), Zn (*p*=0.530), and Mn (*p*=0.563). From the low to the elite group, the average altitude shows a trend of first increasing and then decreasing. Overall, the elite group consists of mostly low-altitude samples but a few high-altitude ones. It demonstrates that the composition and content of different bioactive compounds in plants are clearly regulated by various environmental conditions.

### 3.2 Correspondence analysis of environmental variables and active compounds

**Figure 4:**
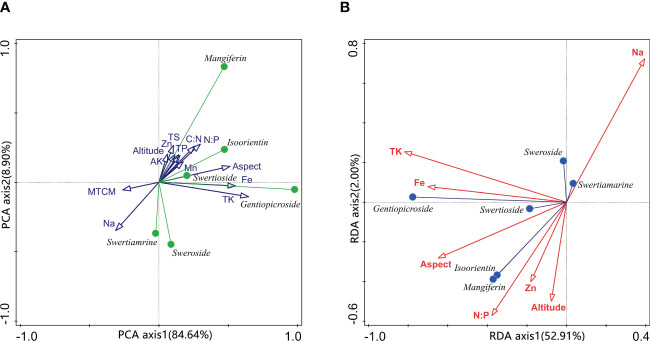
Principal component analysis of environmental variables and compounds-environmental variables biplot of redundancy analysis. TS in **(A)** means total soluble salt content; MTCM means the minimum temperature of the coldest month.

The linear combination of unconstrained ordination analysis for environmental variables has first been used to pre-process the obtained environmental predictors to account for variations in content, which facilitates the classification and contribution of each predictor. As shown in **Figure 4A**, the 13 environmental predictors (see **Table 1**) have been analyzed with the sample chemical type data of bioactive compounds content by unconstrained ordination. In the PCA plots of the two first axes, axis 1 explains 84.64% of all variables and is highly correlated with soil Fe content, aspect, and soil TK content. The direction of axis 1 is also an explanatory dimension for the content of compound B, indicating the contribution of these environmental predictors to this compound. At the same time, the MTCM and Na content present a negative relation with respect to axis 1, leading to an adverse effect. Axis 2 contributes 8.9% of the explanatory power, roughly related to the site soil predictors. Compound D is highly consistent with the direction of these factors, indicating a correlation between them. Since compounds A and C point in the negative direction of axis 2 and are negatively correlated with most environmental factors, this suggests that these two components are only positively correlated with soil Na content and MTCM in the ordination analysis.

To further analyze the correlation between environmental predictors and compound content, constrained ordination analysis has been used to calculate the explanatory variation for 13 environmental predictors. The environmental variables are selected one by one in the forward selection analysis process until the explanatory power exceeded the estimated one in the model by 55.2% and a total of 7 predictors are finally included in the redundancy analysis model. The explained variation of the explanatory variables then reached 55.6%. Axis 1 accounted for 52.91% of the explanation, while axis 2 accounted for only 2%. However, all 4 axes appear to be significant with test F-ratio of 16.126 (*p*=0.002), showing that the redundancy analysis model is remarkably helpful. There were significant differences in the conditional effects of environmental variables, with the soil TK content contributing the most 35.67%. Then there is the aspect, Na, Fe, and Zn content, accounting for 11.3%, 2.7%, 2%, and 1.6%, respectively. The remaining predictors do not stand out in terms of interpretation. As shown in **Figure 4B**, the soil Fe, total K content and aspect of wild *S. mussotii* population growth sites significantly affect the output of compound B. In other words, the more south-facing the habitat site and the higher the site soil total K, the more abundant the compound B in *S. mussotii* plants. Similarly, compounds D and E are strongly correlated with site soil N/P ratio, aspect, Zn content, and altitude. Inversely, the concentration of compounds A and C is significantly promoted by soil Na content.

### 3.3 Structural equation model of bioactive compounds

**Figure 5:**
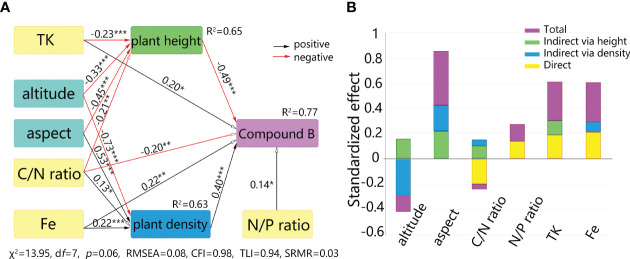
Direct and indirect effects on bioactive compounds of S. mussotii from abiotic factors. **(A)** Structural equation model between bioactive compound and geographical factors. **(B)** Standardized effects from the explanatory variables. * means p<0.05, ** means p<0.01, *** means p<0.001.

Using the wild *S. mussotii* population density and plant height data recorded in the sampling as outcome variables, respectively, the confirmatory factor analysis of the structural equation model has been carried out with environmental predictors using Bootstrap analysis and maximum likelihood estimation. In each fit, the independent variables with the worst significance in the model fit results are excluded and then repeated until the best combination is obtained. With the results for plant height and population density, the different content of bioactive compounds is then incorporated into the structural model. Thus, this structural equation model contains 23 non-collinear environmental predictors as explanatory variables and each bioactive compound content as an outcome variable, mediated by the plant height and population density of *S. mussotii*, and is modified by removing one by one the predictors that did not work well. By permuting the combinations of explanatory variables, the best-fit structural equation model is finally obtained. In the case of compound B, a final set of 6 environmental predictors were used as explanatory variables, including altitude, aspect, C/N ratio, N/P ratio, TK, and Fe content in the soil samples. As **Figure 5** shown, the χ^2^ of the structural equation model is 13.95 with a d*f* of 7, the ratio of which two falls between 1 to 3 (*p*=0.0649), meeting the recommended value. The same holds for the other test values. The CFI and TLI are recommended to be greater than 0.9, while the model values are 0.979 and 0.938, respectively. RMSEA is 0.079 and SRMR is 0.031, both below the threshold value of 0.08. At the same time, a 95% confidence interval test has been performed on the model results *via* Bootstrap analysis, and the returned interval does not contain a zero value for each variable, proving that the estimated values of the explanatory variables are all significant. Briefly, plant height and population density have different negative and positive effects on its output level, respectively. This can be understood as an increase in biological mass within a plant reduces the relative content of bioactive constituents, while growth in dense areas corresponds to higher levels of the content outcome.

As site spatial factors, altitude and aspect have no direct effect on compound accumulation, but have significant indirect effects through plant height and population density, respectively. Altitude has a negative effect on both plant height and population density, followed by two negative direct effects on bioactive content through plant height and one negative indirect effect through population density. As the negative indirect effect is greater than the positive one, the final effect from altitude to compound B manifests as a significantly negative one, -0.13 (*p*=0.023). Likewise, the indirect effect from aspect to compound content is positive and the total standardized effect is 0.43 (*p*<0.001). In terms of site soil factor, the N/P ratio only has a direct effect on the compound B content, while the C/N ratio plays the most complex path of influence. The direct effect of the C/N ratio reaches -0.20(*p*=0.008), and a concurrently indirect effect *via* plant height and density, 0.11(*p*=0.023) and 0.05(*p*=0.032), separately, resulting in a non-significant total effect of -0.04(*p*=0.601). There are both significant direct and indirect effects from soil TK and Fe on compound B output, ultimately resulting in total standardized effects of 0.31(*p*=0.001) and 0.30(*p*<0.001), respectively.

Similarly, results for the other biologically active compounds can be obtained within the structural equation model, as shown in **Table 2**. These environmental factors are here simply distinguished into spatial and edaphic predictors, the former consisting mainly of altitude, aspect, temperature, and precipitation, the latter consisting mainly of soil C/N and N/P ratios, TP, TK, Fe, and Na content, etc. In order to observe the regulation mechanism of different environmental variables, the absolute and superimposed effects of the two factor groups are distinguished. Compounds A, C, D, and E are generally subject to strong spatial factor effects, but the total effect decreases after stacking. This suggests that spatial factors exert a fairly strong effect over the course of the course, but with consequences at different levels depending on the variety of bioactive compound types and combinations of environmental variables. The total effect given by the environmental variables on compounds A, C, and D turn out to be a negative result, suggesting that the same environmental factors have contradictory effects on the accumulation of different bioactive compounds in plants. In addition, compounds D and E receive a much larger absolute effect than B, while the total effect turns out to be smaller than B. This indicates that the different regulatory effects among environmental variables superimpose or counteract each other, resulting in diverse levels of influence effects.

**Table 2:** Standardized effects of environmental predictors on *S. mussotii* compounds.

compounds	spatial		edaphic		total
	absolute	total	absolute	total	
A	1.587	-1.033^a^	0.599	0.233^a^	-0.800
B	0.555	0.303^b^	0.765	0.765	1.068
C	1.168	-0.570^a^	0.152	0.152^a^	-0.418
D	2.120	0.140	1.696	-0.542	-0.403
E	2.018	-0.043	0.366	0.100	0.057
F	0.220	0.220^a^	0.562	-0.008^a^	0.212

a superscript means direct effect, b means indirect effect, no mark means both.

## 4 Discussion

### 4.1 Diversity and heterogeneity of bioactive compound contents

Just as species distribution models have been used to predict where species grow within a study area based on the environmental conditions of several certain locations (Guisan and Thuiller, 2005; Dong et al., 2017), the environmental gradient analysis can also be used to predict the distribution of compound types and their contents under specific environmental conditions, or the optimal environmental conditions required to enrich the types and contents of target compounds (Isah, 2019). This study casts light on the correspondence analysis between target bioactive compounds and growing environmental conditions of wild *S. mussotii* through field sampling and experimental testing and helps to enrich the knowledge of quality improvement, conservation introduction, and equitable utilization of this valuable medicinal plant. Our results demonstrate that metabolomic expression across the geographic distribution of wild-type *S. mussotii* populations can follow a non-linear, non-restrictive, and non-monotonic relationship with environmental variables. It is thus possible to make much greater use of the ecological factors affecting plant SMs production to enhance targeted phytochemicals output (Khare et al., 2020).

Determined by the utility pattern of *S. mussotii* according to medicine application and preparation production, the current research on this medical material mainly focused on its swertiamarin, gentiopicroside, sweroside, mangiferin, and isoorientin content level (Tan et al., 2017). Represented by gentiopicroside of compound B in this study, the active components of *S. mussotii* have been analyzed not by an extensive total metabolomic analysis once samples were collected but by chromatographic separation in the scheme of target natural products (Chen et al., 2015). Research on the quality and standardization of *S. mussotii* has framed the variety of bioactive compounds (Shen et al., 2021). Consequently, the six bioactive compounds focused on in this study have been widely concerned in the existing literature (Yang et al., 2010; Chen et al., 2015; Tan et al., 2017). This study provides unique insight into the many environmental conditions in which these *S. mussotii* compounds are produced. Although county-scale and climatic factors can be viewed as homologous, most wild *S. mussotii* populations grow with variations in the chemical profile under different combinations of spatial and edaphic conditions.

Existing samples can thus be grouped into different levels of content by the K-means clustering method. From poor to abundant, they can be clearly identified into four groups: low, middle, high, and elite. Due to the difference in separation methods, there is no comparability between the samples analyzed in the scheme of this study and the previous results on the absolute contents of bioactive compounds. But the results obtained in this study are in a constituent proportion similar to that reported in former studies conducted in other geographical locations and have told the distinction of wild populations between different sites, like the Sichuan region and Qinghai region (Lu et al., 2009; Yang et al., 2010; Tan et al., 2017). This indicates the diversity of *S. mussotii* chemical types. Subsequent studies can determine whether the 16.3% elite group has the optimal configuration of environmental conditions by excluding the genotype effects. Moreover, if the under-sampled left side of the study region shown in **Figure 1** is supplemented to increase the number of samples, a more accurate and affluent result might be obtained to delve into the details.

The bioactive product content depends on various factors such as plant genetics, harvesting time, crude extraction, and growing environment. Similarly, focusing on different bioactive compounds may yield different results for the same environmental predictor. In this study, a higher amount of compound B was observed under higher TK and Fe edaphic sites and on sunny slopes, leading to a difference between the explanatory variables obtained from its result model and those of the other compounds (see **Figure 2**). On the other hand, the altitude of the spatial factors has a direct and significant effect only on compounds A and C, and aspect has a direct effect on compound F, while both of them have no indirect effect on these three compounds. Altitude also puts an indirect effect *via* plant height to compound D without direct effect, while both direct effect and indirect one *via* population density on compound E. As seen in **Table 2**, compounds A, C, and F mainly suffer from the direct impact of spatial and edaphic factors, while D and E simultaneously impact the direct and indirect effects of environmental conditions. Only compound B stands up to the indirect effects of the spatial factors, while the integrated effects of the edaphic conditions, both of which are positive, lead to the highest level of regulation of the final total effect.

### 4.2 Interaction among geographical factors

With the redundancy analysis and factor analysis in this study, most of the crucial environmental variables that bring about significant changes in the bioactive compounds in wild *S. mussotii* can now be clearly identified as spatial and edaphic factors. The spatial gradients include the aspect, altitude, and minimum temperature of the coldest month, with decreasing explanations and contributions forming a relatively important group of predictors. Given that climate change largely prompts sensitive plants to change the content and composition of SMs by warming (Gérard et al., 2020), the quality and habitats of wild *S. mussotii* may vary greatly under future environmental warming in the climate-vulnerable Qinghai Tibetan plateau. Because higher altitude usually means lower temperature, larger temperature differences, and more solar radiation (Arias et al., 2022). As the overall ambient environmental temperature increases, however, the synergistic adjustment effect between altitude and temperature will gradually change or be lost. This means that existing combinations of geographic factors will adjust in a warm and humid future environmental scenario, leading to variations in bioactive compound content. Thus, there will be a large reduction in the area that can contain *S. mussotii* habitat. Therefore, researchers and related practitioners need to make greater efforts for the conservation and rational utilization of wild *S. mussotii* resources in order to cope with future climate change and human disturbances.

The edaphic factors are composed of TK, Na, TP, Fe, C/N ratio, AK, N/P ratio, Zn, total soluble salt, and Mn content, with the order of the contributions descending in turn. The involvement of so many soil factors suggests that wild *S. mussotii* is strongly dependent on a variety of environmental conditions during its lifetime for the synthesis and accumulation of SMs. However, little literature has reported the tendency of bioactive compound levels of *S. mussotii* across different soil characteristics. For example, soil organic carbon restricts plant-microbial interaction by affecting the communication between plant root flavonoid signal compounds and microbial symbionts, which leads to changes in plant physiological and ecological processes (Del Valle et al., 2020). The regulation of multiple abiotic properties of site soils may also have complex synergistic or antagonistic effects, although soil organic carbon content has been shown to have weak relationships with target compounds, and the biological factors of soil microbial communities have not been introduced in this study. For C/N and N/P ratios, which generally fit the correspondence analysis better in the competing tests, their high relative indicators such as soil organic carbon, TN, and soil pH have been removed from the study. But it is important to point out that the elite group of wild *S. mussotii* populations grows neither in the highest nor lowest soil organic carbon sites, but in the medium soil organic carbon highest TN regions, corresponding to a mid-level of C/N ratio. Besides, soil pH value is highly correlated with conductivity, salinity, and other indicators, and plays a certain role in regulating plant SMs (Kandimalla et al., 2020). The soil pH of 98 sample sites ranges from 7.8 to 9.0, with an average of 8.2, which is just the mean value of the pH index for the elite group. It also shows that the previous general situation described for the physiological demands of the wild *S. mussotii* population is extremely rough (Ji et al., 2020). If more of the growth factors associated with *S. mussotii* are put into a comprehensive and credible framework, then the vital coordination and operation of geographical conditions may be worked out.

After distinguishing the four levels of *S. mussotii* according to the amount of bioactive compounds, the dramatically significant environmental factors are the aspect, the soil TK, and the Na content. In this case, a more southerly slope direction, higher TK, and lower Na content in the soil correspond to a more abundant flora of bioactive compounds. Among them, south-facing means more sunshine, which may be associated with the facilitation that many plants improve SMs outcome under the enhancement of light exposure or UV stress (Amarasinghe et al., 2015; Tohge et al., 2016; Hernández et al., 2022). The effects of different contents of soil total K on the regulation of SMs in plants have also been reported (Liu et al., 2020). The increase in soil Na content may be related to salt stress (Bittencourt et al., 2022). But this study with total soluble salt factor data does not provide a deeper analysis of the correlation between these two indicators but only provides a certain perspective on the plant stress process.

In contrast to the correspondence analysis and the clustering analysis, the structural equation model gives the most detailed and unambiguous correlation between the bioactive compound content and environmental predictors in wild *S. mussotii* populations. The final fitted model explains a mechanism for the influence of spatial factors, namely altitude and aspect, on the bioactive compounds in *S. mussotii* plants. This indirect effect is also consistent with plant physiological metabolism and response to external stress. Previous studies have demonstrated that plant synthesis of flavonoid compounds is synthetically influenced by temperature, light, drought, and salinity in the site habitats. However, this process is not simply expected to improve production by introducing UV radiation or drought stress, but there are highly complex and organized mechanisms related to specific metabolic pathways and enzyme catalysis (Bo et al., 2016; Prinsloo and Nogemane, 2018). There are geographical variations in flavonoids and terpenoids in plant metabolism for altitude conditions that significantly affect the contents of these two compounds and the effects on them are not completely mutually exclusive (Sun et al., 2021). Therefore, an attempt to directly establish a linear or monotonous relationship between different plant populations of *S. mussotii* and altitude conditions may not clearly be obtained (Yang et al., 2010). This study provides a perspective that relationships between the environment and SMs can be mediated by plant physiological and ecological processes, such as plant height and population density. Still, it is undeniable that the indirect effect of plant density from altitude and aspect on the synthesis of bioactive compounds may be mediated by the other factors that also significantly affect population density but were not observed in this study also mediate this indirect effect.

Although the soil C/N ratio does not show a significant total effect or significant results in correspondence analysis, it has a statistically quantifiable indirect effect on the bioactive compound content of the *S. mussotii* plant. Since the C/N ratio of available samples is relatively low, the correspondence analysis model does not identify the arched effect on the SMs content of *S. mussotii* caused by superfluous nutrition, that is, the process of first increasing and then decreasing with the increase of total carbon and nitrogen contents (Scogings, 2018). In fact, long-term nitrogen and phosphorus addition changes the carbon distribution of plants to carbon-based defense compounds, showing a close correlation between SMs content and plant carbon, nitrogen, and phosphorus stoichiometry (Shan et al., 2018). Thus, the present results can only demonstrate the plasticity of the bioactive output in *S. mussotii* plant growing in poor conditions of soil carbon and nutrient content. Further relation needs to be supplemented with data under diverse nutritional conditions.

In the end, correlation and significance in statistics do not imply causal links in reality, as the knowledge available to correlate environmental variables of *S. mussotii* with changes in bioactive compounds is based on ordination analysis and regression models. Attributes such as soil types, thickness, and granularity size compositions, as well as *in situ* control tests, all need to be checked before large-scale conservation introductions could be formally undertaken (Yun et al., 2018). While the spatial factors which have a distinct effect on the quality of raw *S. mussotii* material cannot be neglected. Thus, human intervention and habitat conservation may remain the most appropriate coping strategies at this stage. As one local guide put it, the demand for wild plant resources has increased in recent years, but the awareness and adaptation measures for their sustainable use have not kept pace. The one act of ability which he deemed meaningful and mindful of his duty was to sow the seeds of these ripening herbs in the places where they had been growing. This is also the purpose and motivation for this study.

## 5 Conclusion

This study has analyzed the correlation between the amount of bioactive compounds and environmental variables for wild *S. mussotii* populations. The content of biologically active compounds varies with different environmental conditions, and a change in an individual factor may alter the SMs content. We report on the variability of the main bioactive compounds in *S. mussotii* material sampled from Xiaojin County in western China and demonstrate the existence of an elite group with the optimal combinations of geographical conditions and the highest bioactive compound content. Key factors associated with the high quality of wild *S. mussotii* populations include the altitude, aspect, air temperature and solar radiation intensity, soil TK, Fe content, and C/N and N/P ratios. The standardized effects and the final stacking result differ significantly between the two sets of abiotic factors, so the regulation of plant SMs by environmental variables should be analyzed in terms of the combination of target compounds and geographical predictors.

In view of the fact that spatial factors have a non-negligible effect on herb quality, it is necessary to consider similar habitats in the practice of remote introduction. This knowledge provides an explanatory basis for further laboratory and field control experiments to identify the underlying environmental factors that enhance the quality of this valuable medicinal plant. There may be many more other factors that affect the bioactive ingredient content, such as endogenous genetic and exogenous abiotic and biotic factors, as well as the collection, vegetation, and storage of plant material itself. In addition to examining the genetic differences among *S. mussotii* populations at different sample sites, future studies need to systematically design a more comprehensive framework for plant SMs formation and accumulation and various combinations of environmental conditions, as well as plant-environment interactions, to finally confirm the optimal configuration of environmental conditions to improve medicinal quality.

## Data availability statement

The datasets generated for this study are available on request to the corresponding authors.

## Author contributions

XW and CS contributed equally to this work. XW and YL: conceptualization. XW, CS, XZ and YL: validation. XW, TC and CS: investigation. XZ, TC and CS: experiment and analysis. XW and CS: writing-original draft preparation. CS, TC, XZ and YL: writing-review and editing. XW, TC and YL: funding acquisition. All authors contributed to the article and approved the submitted version.
